# Gut microbiome signature of Viliuisk encephalomyelitis in Yakuts includes an increase in microbes linked to lean body mass and eating behaviour

**DOI:** 10.1186/s13023-020-01612-4

**Published:** 2020-11-20

**Authors:** Veronika Kuznetsova, Alexander Tyakht, Lyudmila Akhmadishina, Vera Odintsova, Natalia Klimenko, Elena Kostryukova, Maria Vakhitova, Tatyana Grigoryeva, Sergey Malanin, Vsevolod Vladimirtsev, Raisa Nikitina, Viktor Volok, Vladimir Osakovskiy, Tatiana Sivtseva, Fyodor Platonov, Dmitry Alexeev, Galina Karganova

**Affiliations:** 1 Center for Precision Genome Editing and Genetic Technologies for Biomedicine, Institute of Gene Biology of Russian Academy of Science, Vavilova Str. 34/5, 119334 Moscow, Russian Federation; 2 Moscow Institute of Physics and Technology, Kerchenskaya Str. 1A, Moscow, Russian Federation 117303; 3 Atlas Biomed Group - Knomics LLC, Tintagel House, 92 Albert Embankment, Lambeth, London SE1 7TY UK; 4 Sechenov First Moscow State Medical University, Trubetskaya Str. 8-2, 119991 Moscow, Russian Federation; 5 Federal Research and Clinical Centre of Physical-Chemical Medicine of Federal Medical Biological Agency, Malaya Pirogovskaya Str. 1a, 119435 Moscow, Russian Federation; 6 Institute of Fundamental Medicine and Biology, Kazan Federal University, K. Marx Str. 18, 420012 Kazan, Russian Federation; 7 Research Center of Medical Institute, M.K. Ammosov North-Eastern Federal University, Belinsky Str. 58, 677027 Yakutsk, Russian Federation; 8 Chumakov Institute of Poliomyelitis and Viral Encephalitides (FSBSI “Chumakov FSC R&D IBP RAS), prem. 8, k.17, pos. Institut Poliomyelita, poselenie Moskovskiy, 108819 Moscow, Russian Federation; 9 ITMO University, Kronverkskiy pr. 49, 197101 Saint-Petersburg, Russian Federation

**Keywords:** Gut microbiome, Viliuisk encephalomyelitis, Yakuts, 16S rRNA sequencing, *Methanobrevibacter*, Microbiome-gut-brain axis

## Abstract

**Background:**

Viliuisk encephalomyelitis (VE) is a rare endemic neurodegenerative disease occurring in the Yakut population of Northeastern Siberia. The main clinical features of VE are spasticity, dysarthria, dementia, central paresis and paralysis, and cortical atrophy observed via MRI. Many hypotheses have been proposed regarding its etiology, including infectious agents, genetics, environmental factors, and immunopathology. Each of these hypotheses has been supported to some extent by epidemiological and experimental data. Nevertheless, none of them has been decisively proven. Gut microbiome is one of the factors that might be involved in VE pathogenesis.

**Results:**

Here we performed a pilot survey of the stool microbiomes of Yakut subjects with VE (n = 6) and without VE (n = 11). 16S rRNA sequencing showed that in comparison with the control group, the Yakuts with VE had increased proportions of *Methanobrevibacter* and *Christensenella*, which are reported to be linked to body mass index, metabolism, dietary habits and potentially to neurodegenerative disorders. The identified associations suggest that the microbiome may be involved in VE. Overall, the Yakut microbiome was quite specific in comparison with other populations, such as metropolitan Russians and native inhabitants of the Canadian Arctic.

**Conclusions:**

Describing the gut microbiome of indigenous human populations will help to elucidate the impact of dietary and environmental factors on microbial community structure and identify risks linked to the lifestyles of such groups as well as endemic diseases.

## Background

Viliuisk encephalomyelitis (VE) is a unique progressive disorder of the nervous system that affects Yakuts and is especially prevalent among Yakut inhabitants of the Viliui river valley [1]. It was initially described by R. A. Maack in 1854 during an expedition to the Yakut region. of the disease occurred in several regions in the Viliui river valley in the 1950s and subsequently spread into central regions of Yakutia in the 1970s. During these decades, more than 2′000 cases were registered. Later, there was observed a decline in the incidence of VE. New cases were registered mostly in people living in or originating from several districts alongside the Viliui river, and to a lesser extent, in Yakuts from other territories. Currently, only about 20 patients, who were followed for 10–40 years, are living with the diagnosis of VE. The observed reduction in the incidence of VE apparently reflects the real decline of the disease, although social factors, such as the reluctance of patients and their families to draw attention to their disease in order to avoid stigmatization, and the decline in the awareness of VE among medical professionals, may also play a role.

A detailed clinical profile of VE including its diagnostic criteria and pathophysiological mechanisms is described in [1, 2]. The main clinical features of VE are spasticity, dysarthria, dementia, central pareses and paralysis, and cortical atrophy observed via MRI. VE has three forms: acute, subacute and chronic [1]. The acute form VE is accompanied by fever, severe headache, impaired consciousness, meningoencephalitis and pyramidal damage. The disease progresses rapidly and often leads to a fatal outcome within 12 months. Subacute form of VE progresses from 1 to 6 years and is characterized by meningoencephalitis with the subsequent development of dementia, dysarthria, damage to the pyramidal and extrapyramidal systems, and diffuse brain atrophy. The chronic form of VE proceeds either in the form of diffuse brain damage with the development of spastic pareses and dementia, or with a development of spastic para- or tetraparesis without a significant deterioration in cognitive functions. Chronic VE is distinguished by long periods of stabilization of symptoms and lasts for more than 6 years. While the acute and subacute forms of VE are characterized by an active inflammatory process and lymphocyte infiltration in different structures of the CNS, sclerotization is observed in the chronic form [1–3]

While the outbreak in the 1970s received significant attention, efforts to discover the nature of VE were unsuccessful [3]. Since the first reports of the disease, many hypotheses have been proposed regarding its etiology, including infectious agents [2], genetics [1, 4], environmental factors, and immunopathology [5, 6]. Each of these hypotheses has been supported to some extent by epidemiological and experimental data. Nevertheless, none of them has been decisively proven. Although VE tends to aggregate in families, cases of the disease were observed in adopted children genetically unrelated to their host families [5]. The search for infectious or environmental causative agents provided some intriguing insights but eventually proved futile [2]. Considering the recent unexplainable decline in VE morbidity [7], the causes of this unique and severe disease could remain unknown, heightening the need for more comprehensive research.

The gut microbiome is a crucial factor in sustaining homeostasis, barrier function, immune system development, and metabolism for humans. The impact of commensal microbes is not limited to the gastrointestinal tract but rather encompasses all organs. Alterations in microbiome composition can be caused by various environmental stressors like dietary changes, medication intake, infections or lifestyle changes. These alterations can also include markers of pathological states or reveal certain steps of pathogenesis for diverse metabolic, neurological, immune and neoplastic disorders [8–15].

Neurological disorders are of particular note, including neurodegenerative diseases. Associations have been found between altered gut microbial community structures and Parkinson’s disease [8], multiple sclerosis [9, 10], autism [11], Alzheimer’s disease [12], Huntington’s disease, amyotrophic lateral sclerosis and neuromyelitis optica spectrum disorder [13]. These associations are supported by experimental models [14, 15] that allow the expansion of the concept of the “gut–brain axis” in the pathological context to include the microbiome as an additional element. In one mechanism, aggregated alpha-synuclein acts as a messenger that alerts the immune cells in the central nervous system (CNS) to the presence of certain pathogens, with bacterial exposure as a potential driving force behind alpha-synuclein aggregation in Parkinson’s disease [16]. Another example is the direct participation of the oral microbe *Porphyromonas gingivalis* in the synthesis of substances that contribute to the development of Alzheimer’s disease [17].

Previous studies have profiled the gut microbiomes of geographically diverse ethnic groups transitioning from their native ways of life to Westernized lifestyles and diets, including the Hadza, Inuits, Arapaho, Cheyennes, Burkina-Faso and Malawi [18–22]. Differences in microbial composition between agricultural and urban populations as well as unique compositional characteristics present in hunter-gatherers have been identified [18]. Notably, the same microbial genera were shown to play different ecological roles in the guts of subjects from different regions [23].

The health problems of isolated populations are often exacerbated by difficult climatic and socioeconomic circumstances including household food insecurity [24] and can also be linked to endemic or globally occurring diseases. For example, gut dysbiosis was associated with metabolic disorders in Arapaho and Cheyennes. Such observations highlight the importance of searching for potential disease biomarkers in the microbiomes of ethnic groups and isolated populations. Moreover, such biomarkers might provide clues for improving healthcare not just for specific populations but also on a global scale. A better understanding of isolated populations’ gut microbiomes could both improve healthcare practices and reveal beneficial microbes that have been lost or are on the verge of extinction in humans.

Yakuts are a Turkic ethnic group with a population of 466,492 (as of 2010) inhabiting the Sakha Republic (Yakutia) in Northeastern Siberia, the largest and coldest region of Russia. Available historical and genetic data suggest that Yakuts are direct descendants of Turkic nomads who migrated from Mongolia and the Altai-Sayan region around the 11th–thirteenth century [25]. Large-scale analysis of Y-chromosomal and mitochondrial haplogroups combined with autosomal SNPs has shown very low diversity in the male gene pool (“the founder effect”) with evidence of recent gene flow between Evenks and Yakuts [26]. The role of natural selection in establishing the population’s adaptation to the cold climate has been emphasised [27]. The photoperiod of the Yakut region ranges from 5 to 20 h per day.

The Yakut diet is unstable and scarce [28]. Yakuts and other ethnic groups inhabiting Sakha consume a traditional diet rich in animal fats and meat. Exome analysis has shown polygenic mechanisms underlying adaptations to this diet [29]. Recently, due to the development of transport infrastructure in the region, the average Yakut diet has approached the typical Russian diet and is insufficiently balanced in terms of protein, fat, carbohydrates, micronutrient content and calories [30]. The observed transformations mirror the substitution of traditional diets with the “Western diet” observed in North American populations such as the Inuits; in the case of the likely explaining the similarity of the Inuit microbiome profile to that of Western populations [31].

Since VE has many clinical and pathophysiological similarities to other diseases of the nervous system, it is reasonable to suspect that the gut microbiome is involved in its onset or progression. The VE-associated microbiome could contain microbial agents that are directly involved in pathogenesis. Also, a dysbiotic gut community could lead to disturbances of the immune response with possible neurodegenerative consequences.

Considering the increasing evidence of the importance of the microbiome–gut–brain connection and the wide gaps in knowledge about the microbiome composition of populations relatively unaffected by globalisation, it is worth investigating the potential role of the microbiome as a causal or aggravating factor of VE.

## Methods

### Study design and sample collection

The study was performed as a part of the project “Discovering molecular genetic pathogenetic mechanisms of chronic degenerative pathologies in Yakutia: Viliuisk encephalomyelitis, amyotrophic lateral sclerosis, chronic viral hepatitis” conducted by the Federal State Budgetary Institute Yakut Research Center of Complex Medical Problems of the Siberian Branch of the Russian Academy of Medical Sciences #167.

Initially, 21 subjects were enrolled in the study (14 females and seven males, aged 22–71), of whom 14 did not have chronic VE, six had diagnosed VE, and one was suspected of having VE. The diagnosis of chronic VE was based on the clinical criteria described in detail in [1] and [2]. Participants completed a questionnaire including information about chronic diseases, medication intake and other factors. Patients diagnosed with definite or suspected chronic VE were examined by a neurologist, and the clinical data are presented in Additional file 1: Table 1. A single female patient had suspected acute VE in 2009, a family history of VE, and moderate residual neurological disturbances but could not be definitively diagnosed. Thus, she was included in the VE risk group and eventually excluded from the analysis on the grounds of oral contraceptive use.

**Table 1:** Gut microbes associated with Viliuisk encephalomyelitis

Taxon	Level	Coefficient	p-value	Adjusted p-value
*(a) Positive associations*				
p__Euryarchaeota	Phylum	2.087740	0.000289	0.002313
c__Methanobacteria	Class	2.087740	0.000289	0.004048
o__Methanobacteriales	Order	2.087740	0.000289	0.005783
f__Methanobacteriaceae	Family	2.087740	0.000289	0.020698
g__Methanobrevibacter	Genus	2.133336	0.000226	0.012647
o__Clostridiales;f__	Family	1.205089	0.000773	0.014298
g__Methanobrevibacter;s_	Species	2.133336	0.000226	0.015809
o__Clostridiales;f__;g__	Genus	1.205089	0.000773	0.021640
o__Clostridiales;f__;g__;s__	Species	1.205089	0.000773	0.027050
*(b) Negative associations*				
g__Streptococcus;s__	Species	− 1.928370	0.001641	0.038280

Significance threshold: adjusted p < 0.05

In an attempt to encompass a general population sample in this pilot study, the inclusion and exclusion criteria were minimal: for the experimental and control groups, the main criteria were the presence or absence of a VE diagnosis, correspondingly. The presence of any acute disease at the moment of enrolment was an exclusion criterion for both groups. After adjustment (see Results), the final VE group had six subjects (three males and three females, aged 48–71), and the control group had 11 subjects (four males and seven females, aged 26–60).

Stool samples were collected using sterile vials. The samples were stored at −20 °C after transportation for no longer than six hours at 4 °C. Further handling was performed with refrigerants to prevent thawing.

### Sample preparation and microbiome sequencing

DNA extraction from the stool samples was performed as described previously [32]. The V3-V4 regions of the 16S rRNA gene were amplified. High-throughput sequencing was performed using an Illumina MiSeq platform yielding more than 20,000 reads per sample.

### Bioinformatic analysis

The data were analysed in the Knomics-Biota system [33] on forward (R1) reads. The “basic report” feature was used for quality control and preprocessing (all samples were rarefied down to 3,000 classified reads). The reads were denoised with the Deblur algorithm [34]. Further taxonomic assignment based on the GreenGenes database v13.5 [35] with a Naive Bayes classifier was implemented in QIIME2 [36].

The “factor report” feature was used to identify statistical links between microbiome composition and experimental factors. A generalised mixed-effects linear model with a negative binomial distribution family of residuals was fitted for each taxon to identify associations with clinical status (VE-positive or VE-negative). The model included antibiotic use as a random effect. Rare taxa were excluded from the analysis (taxa were included if present in at least 10% of the samples at the level of > 0.2%). A multiple testing adjustment was performed using the Benjamini–Hochberg procedure.

We compared the Yakut microbiome with published data on metropolitan Russians [37] and Inuits [31] using the “external comparison report” feature of Knomics-Biota via closed-reference picking with the GreenGenes database v13.5 [35] and QIIME 1.9 [38].

### Compositionality-aware statistical analysis

We performed a complementary compositionality-aware analysis of the association of microbiome composition and VE. In this analysis, the original read count table was analysed with the selbal algorithm [39]. The algorithm investigates the association between microbial balance—the normalised log-ratio of the bacterial abundances’ geometric means for two groups of bacteria (numerator and denominator)—and an experimental factor. The algorithm includes three steps: (1) estimation of the optimal number of predictors for the investigated factor, (2) identification of the best balance for factor prediction from all possible numerators and denominators and (3) estimation of the numerator and denominator members’ stability using cross-validation. The analysis included only microbes that were present in at least five samples at > 1% levels.

We considered an association significant if the p-value from the linear regression analysis between the balance and the factor was < 0.05 and the taxon was selected as a part of the balance in > 20% of cross-validation iterations. This analysis did not include an adjustment for antibiotic use due to the small sample size.

## Results

### Cohort description

The samples were collected in March 2015 from donors from several community types: eight subjects from a city (Yakutsk, population 311,000), 11 subjects from a town (Viliuisk, population 11,000, including both urban and rural dwellings) and two rural subjects. The study was descriptive, as the sample represented the general population with minimal inclusion–exclusion criteria. The participants’ age was 22–71 years, including 14 women and seven men with normal body mass indices (BMI; within the range of 25 to 35). There was a trend of difference in age of the subjects between the VE group and the control group (Mann–Whitney U test, p = 0.05589).

According to self-administered questionnaires, four subjects reported at least one condition or factor that could strongly affect their microbiome composition but could not be adjusted for during the statistical analysis due to the low number of subjects and unbalanced sampling. These conditions and factors included a gastric ulcer (in remission), pancreatectomy or cystectomy, hepatitis C, chronic obstructive pulmonary disease (COPD), intake of nonsteroidal anti-inflammatory drugs (NSAIDs), pregnancy, oral intake of contraceptives and type 2 diabetes. These subjects were excluded from the analysis. One subject who was excluded due to the oral intake of contraceptives was the only subject belonging to a VE risk group. She had suspected acute VE in 2009 and had some residual neurological symptoms that could indicate possible chronic progression in the future, but overall she did not meet the established diagnostic criteria of VE.

Eight subjects reported they had received antibiotics within the month prior to the study. Many of the subjects reported chronic pancreatitis (n = 7), chronic pyelonephritis (n = 9) or hepatitis B (n = 6). In total, the analysed cohort included 17 subjects (10 females and seven males) including six patients with VE.

### Characteristics of Yakut gut microbiome

**Fig. 1:**
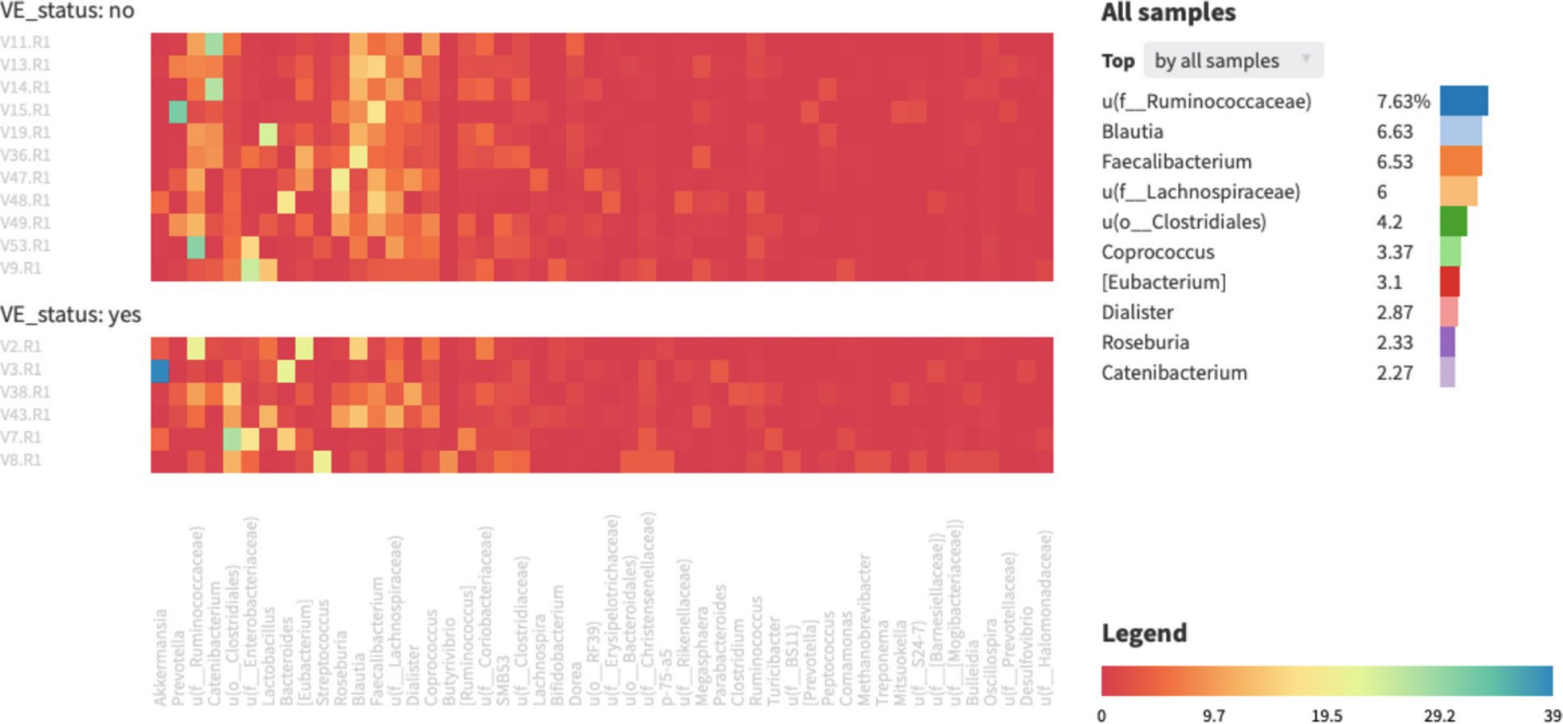
Gut community structure of Yakuts. Heatmap of the relative abundance of major microbial genera in the Yakut gut microbiome (snapshot of an interactive visualisation in the Knomics-Biota online system). The two subplots correspond to control subjects (n = 11) and Viliuisk encephalomyelitis patients (n = 6). The bar plots on the top right show the levels of the ten most-abundant taxa averaged across all subjects

Taxonomic profiling of stool samples from the entire cohort via 16S rRNA gene sequencing data detected 90 genera in 49 families (major genera shown in Fig. 1).

**Fig. 2:**
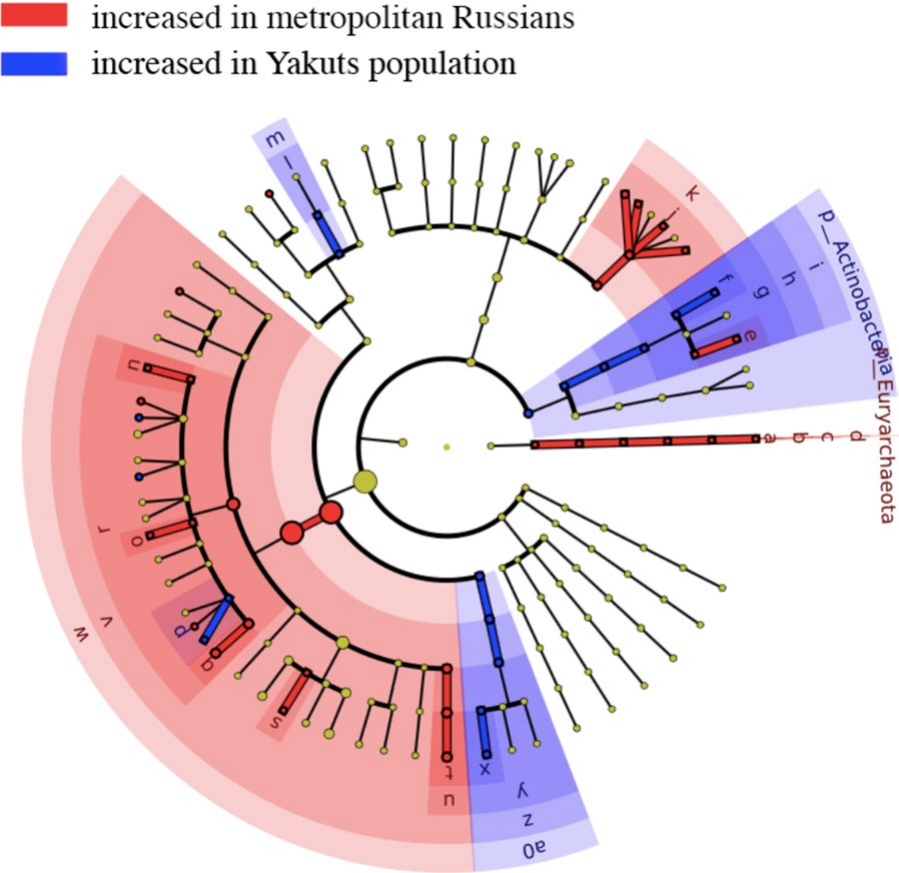
Cladogram highlighting differences in the abundance of microbial taxa in Yakuts without Viliuisk encephalomyelitis (n = 11) and metropolitan Russians (n = 101; external data comparison). The analysis was performed using a generalised linear model (GLM); significance threshold: FDR-adjusted p < 0.05. Notation “g__u(f__Coriobacteriaceae)” refers to “one or more unclassified genera from *Coriobacteriaceae* family” and so on

To identify the unique features of the Yakut gut microbiome, we compared the healthy Yakut microbiome profiles with those of residents of Moscow. The Moscow residents were participants of the crowd-funded OhMyGut project linking microbiome characteristics with diet and lifestyle [37] (n = 101 subjects; only the baseline microbiome profiles before the dietary intervention were considered). Alpha-diversity was lower in the urban residents (p = 0.0). As the 16S rRNA region was different between the two studies, the comparison was performed at the level of genera rather than species. The differences were quite pronounced, including 11 genera with significantly increased abundance in Yakuts and nine with decreased abundance in Yakuts (generalised linear model, adj. p < 0.1; the complete list of differences is described in Additional file 2: Table 2; see also Fig. 2). Particularly, Yakuts had higher levels of *Lactobacillus* represented by *Lactobacillus ruminis*, which was undetectable in metropolitan dwellers.

**Table:** 

	Yakuts		Metropolitan Russians
**f**	g__u(f__Coriobacteriaceae)	**a**	g__Methanobrevibacter
**g**	f__Coriobacteriaceae	**b**	f__Methanobacteriaceae
**h**	o__Coriobacteriales	**c**	o__Methanobacteriales
**i**	c__Coriobacteriia	**d**	c__Methanobacteria
**l**	g__Lactobacillus	**e**	g__Adlercreutzia
**m**	f__Lactobacillaceae	**j**	g__Bacteroides
**p**	g__[Ruminococcus]	**k**	f__Bacteroidaceae
**x**	g__Catenibacterium	**n**	g__Anaerostipes
**y**	f__Erysipelotrichaceae	**o**	g__Lachnobacterium
**z**	o__Erysipelotrichales	**q**	g__u(f__Lachnospiraceae)
**a0**	c__Erysipelotrichi	**r**	f__Lachnospiraceae
		**s**	g__Oscillospira
		**t**	g__u(o__Clostridiales)
		**u**	f__u(o__Clostridiales)
		**v**	o__Clostridiales
		**w**	c__Clostridia

### Universal signature of indigenous Northern microbiome: comparison with inuits

The lifestyle of Northern Canadian Inuits and the Yakut lifestyle share several characteristics that affect the microbiome. The Inuit diet is high in low-carbohydrate products that are rich in animal fat and protein, while Western diets typically involve the regular intake of industrially processed food. Moreover, both populations inhabit regions characterised by cold winters and permafrost.

**Fig. 3:**
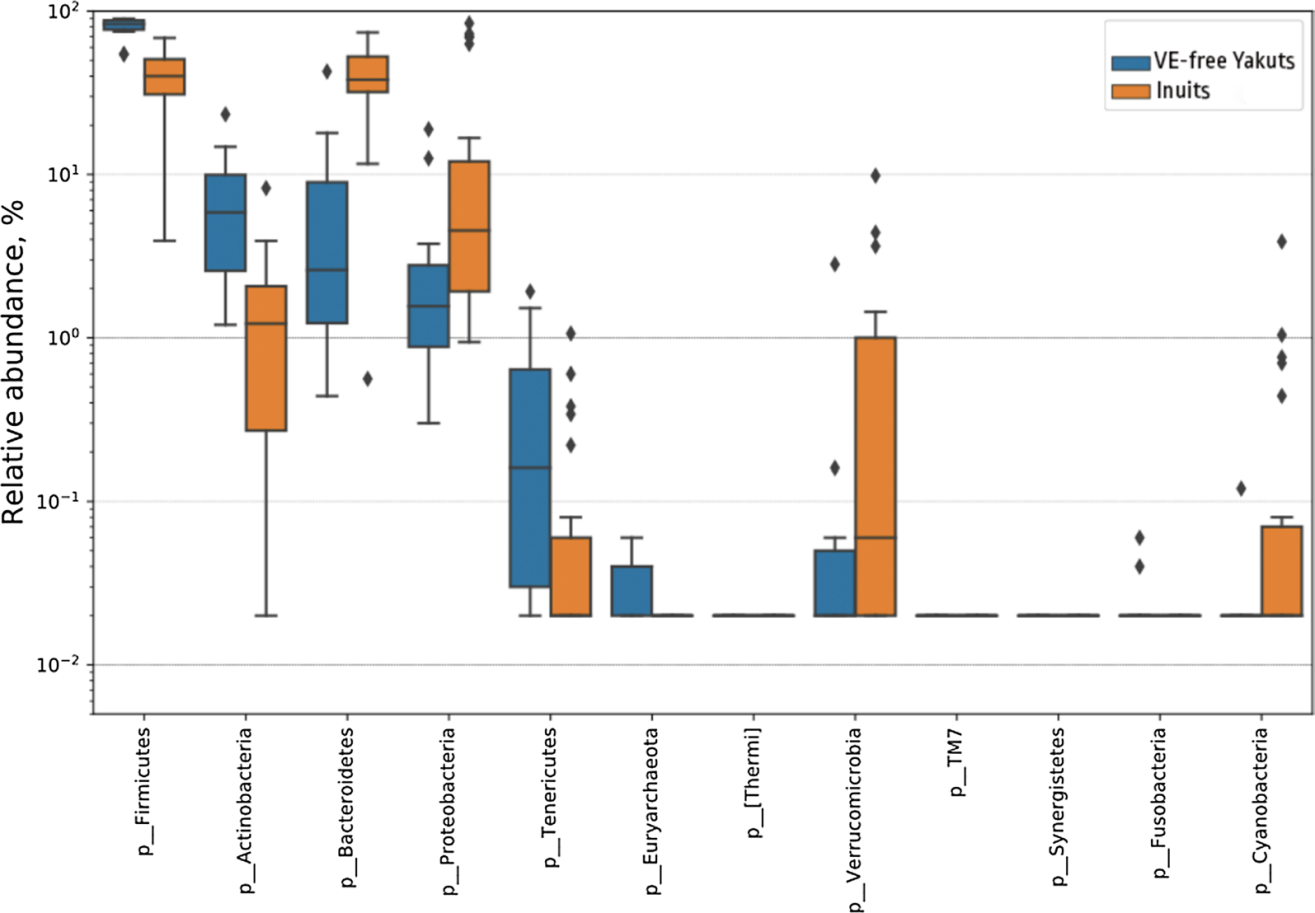
Major microbial phyla compared in abundance between VE-free Yakuts (n = 11) and Inuits (n = 22). The phyla significantly differentially abundant in Yakuts were: increased—*Firmicutes* and *Actinobacteria*; decreased—*Proteobacteria* and *Bacteroidetes* (GLM, FDR-adjusted p < 0.05)

In light of these similarities, we compared the microbiome compositions of VE-free subjects from our study with previously published data on Inuits (22 subjects from the Resolute population in the Canadian territory of Nunavut) obtained using a similar approach [31]. Interestingly, stark differences were noticeable even at the phylum level. Inuits have higher levels of *Bacteroidetes* and *Proteobacteria*, and lower levels of *Firmicutes* and *Actinobacteria* (Fig. 3); as expected, there were even more differences at lower taxonomic ranks (not shown).

### Links of microbiome with Viliuisk encephalomyelitis

We investigated associations between gut microbiome composition and VE using a generalised linear mixed-effect model. Comorbidities and medication intake can independently affect the gut microbiome profile [40]. The proportion of subjects who had taken antibiotics was not significantly different between the VE and control groups, although the proportion was high overall (three of 6 subjects with VE and three of 11 without; Fisher’s exact test, p = 0.6). The intake of antibiotics can be considered a heterogeneous factor; while some types of antibiotics affect specific microbial clades, several participants reported that they had taken multiple antibiotic types or did not provide details about the type. Therefore, in the statistical model, the antibiotic intake factor was treated as a random effect. The main factor of interest was the presence of VE.

Many subjects reported pancreatitis, pyelonephritis or hepatitis B. Each of these diseases can influence the composition of the microbiome. However, VE was not significantly correlated with any of these diseases in our cohort (precise Fisher test, p = 1). Due to their lack of correlation with VE and the study’s small sample size, we did not include these diseases as factors in the statistical model.

We found that VE was significantly associated with changes in microbiome composition after adjusting for antibiotics intake. We observed a trend towards the shift of overall community structure in VE patients (PERMANOVA, adj. p = 0.082846). At the taxa level, the VE group had significantly higher levels of the archaeon *Methanobrevibacter*, which is represented by *M. smithii* in the human gut, as well as certain members of *Clostridiales* order (FDR-adj. p-value < 0.05, see Table 1). At the verge of significance, we observed increase of *Christensenellaceae* and *[Mogibacteriaceae]* families (adj. p = 0.095782 and 0.074482, respectively). Alpha-diversity was not significantly different between Yakuts with and without VE (Wilcoxon rank-sum test, p = 0.871).

**Fig. 4:**
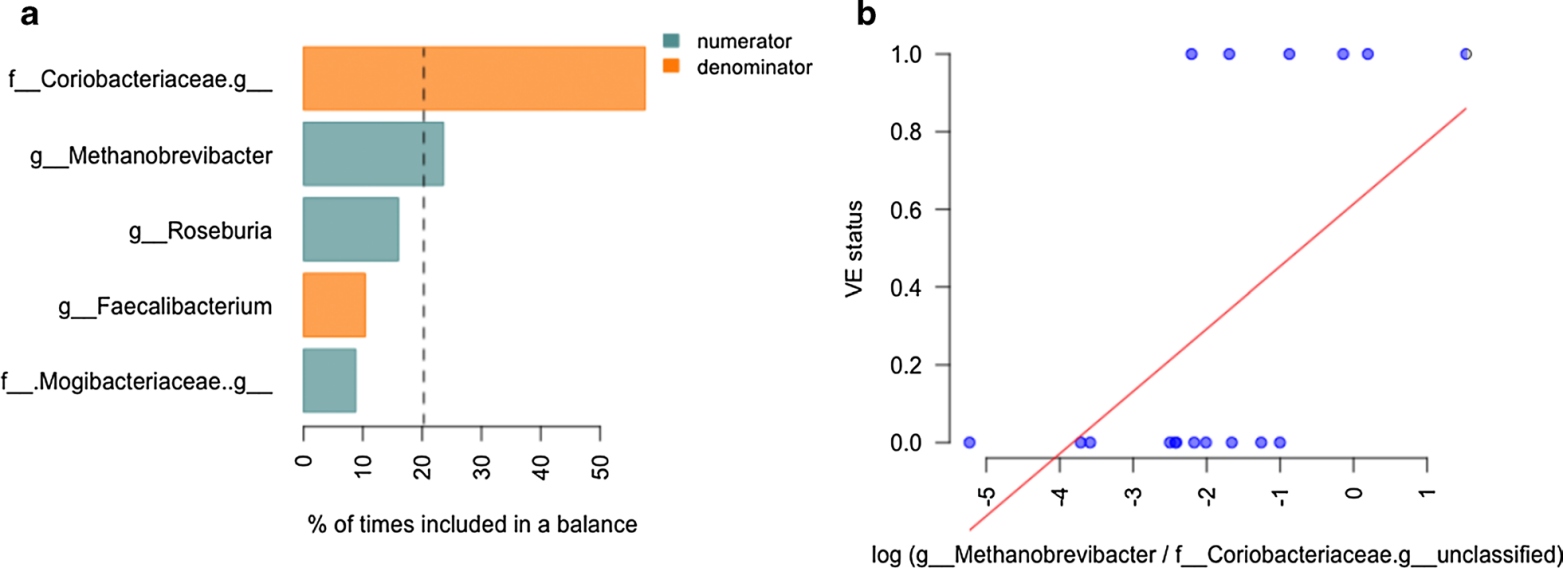
Microbes significantly linked to Viliuisk encephalomyelitis according to the selbal method. **a** The taxa that were included in three most frequent balances during cross-validation procedure. For each taxon, the percent of times it was included in balances during cross-validation is shown. **b** The relationship between the balance selected for the full dataset and the outcome (VE status)

To look for associations between the microbiome and the disease from an alternative perspective, we applied a compositionality-aware selbal method (see “Methods”). This analysis showed links between *Methanobrevibacter* and VE. According to the selbal algorithm, there was trend for association of the disease with the normalised log-ratio (balance) between *Methanobrevibacter* and one or more unclassified genera from the *Coriobacteriaceae* family (p = 0.0539, linear regression). The stability of the balance members was relatively high: *Methanobrevibacter* was selected as one of the best predictors in 23.6% of cross-validation iterations, and the unclassified *Coriobacteriaceae* were selected in 57.6% (Fig. 4).

## Discussion

Viliuisk encephalomyelitis is a rare regional neurodegenerative disease of unknown etiology. Since microbiome surveys of local populations could help to elucidate potential biomarkers of rare diseases and identify mechanisms of their onset and development, our pilot study was aimed to assess whether there are shifts in the microbiome composition in patients with VE. Primarily, we considered the description of the gut microbiome profile of Yakuts in order to better understand the global landscape of gut microbiome diversity. An initial analysis of clinical data revealed a set of comorbidities for a high proportion of the participants. Since this observation likely reflected the population’s socioeconomic and climatic conditions, subjects with prevalent comorbidities were included in the analysis.

Notably, many subjects reported recent antibiotic use. Obviously, this factor alone can alter microbiome composition. To adjust for the effect of antibiotics, we introduced “antibiotic intake” during the assessment of the microbiome signature of VE in Knomics-Biota system. The antibiotic intake factor was included not as a binary but rather as a random effect (flag “factor: to adjust for” in Knomics-Biota metadata configuration).

As an additional analysis, we compared the two populations with previously published data from other samples: first, from large cities in the same country and second, from another country with similar dietary and living conditions.

Our previous general comparison of the gut microbiomes of rural and urban populations in Russia did not reveal strong differences [41]. Here, we found that the Yakut microbiome had characteristic shifts in the relative abundance of several clades. Particularly, the major taxa enriched in Yakuts included members of Actinobacteria (*Coriobacteriaceae* family, 3.8 ± 2.6% in Yakuts vs 1.7 ± 1.4% in Moscow dwellers) and Firmicutes (including *Erysipelotrichaceae* family, 9.1 ± 7.7% vs 2.2 ± 2.2% and *Lactobacillus,* 3.0 ± 5.8% vs 0.1 ± 0.3%).

Strikingly, the *Lactobacillus* in Yakuts was dominantly represented by a single species—*Lactobacillus ruminis*—a non-typical representative of the genus with unique characteristics. The identity of the sequence was confirmed via an amplicon sequence variant (ASV) analysis. *L. ruminis* has been reported to be autochthonous (i.e. a native species of a particular region) and is the only known *Lactobacillus* isolated from mammals with a motile phenotype and immunomodulatory activity [42]. The members of the *Erysipelotrichaceae* family (represented by *Catenibacterium* in Yakuts) are linked to immunogenicity, but their role is unclear [43]. Two of the 17 Yakuts harboured fibre-degrading *Treponema* representatives, which is a known feature of the non-urban microbiome [44]. While *Treponema* has been detected previously only in Southern populations, our observations suggest that it is a cross-climatic feature of the indigenous microbiome. Overall, microbiome richness (alpha-diversity) was significantly higher in Yakuts than in Moscow citizens. The observed differences can be attributed to variations in climate, lifestyle, and diet (Yakuts consume more raw frozen meat and fewer fruits and vegetables than Russia’s urban populations). The possible effect of some technical factors cannot be completely ruled out, including variations in the choice of 16S rRNA gene region (V3-V4 in the present study vs V4 for the Moscow study) and the sample preparation protocol. Still, the enrichment of some immunogenic taxa in the Yakut gut compared to the urban population suggests that the microbiome may have a stronger effect on immunity education in Yakuts. We suggest that the unique community structures with high immunogenic potential observed in indigenous populations are a potential source of novel probiotics and faecal mass transplants (FMT). Such transplants could prevent diseases with autoimmune and allergic components that are prevalent in Westernized countries.

The co-occurrence analysis of microbial genera revealed another feature specific to the Yakut microbiome: the presence of an original cluster of highly correlated low-abundance microbes including *Nesterenkonia*, *Halomonas*, unclassified member(s) of *Comamonadaceae* and others. Although members of this cluster have been previously observed in the human gut [45, 46], its presence could be due to experimental contamination. Notably, this low-abundance cluster does not affect the overall conclusions of our analysis.

The comparison of Yakuts with Inuits revealed even greater differences in microbiome composition than with the Moscow population. This observation was quite unexpected, as Yakuts and Inuits have much in common in their dietary habits. Animal fat and protein are prevalent in both diets, along with low consumption of fruit and vegetables (Inuits might have more fish and fat in their diet). The differences were noticeable even at the level of microbial phyla: Yakuts had a significantly lower *Bacteroidetes*:*Firmicutes* (B:F) ratio (0.107 vs 1.042 in Inuits on average). Interestingly, this is consistent with the previous microbiome meta-analysis showing that it is one of the key features distinguishing the urban populations of Russia and the United States [32]. Although some of the differences may reflect variations in experimental protocol, we observed evidence of intercontinental differences in phylum-level gut microbiome composition.

Further, we compared the gut microbiome composition of Yakuts with and without VE. The microbiome signature of VE observed at various taxonomic levels includes an increase of *Methanobrevibacter* and, with lower significance, *Christensenellaceae*. Several commensal clostridia were reduced in the VE group. *Methanobrevibacter* with its representative species *M. smithii* is a major human gut archaeon. The taxon has been associated with BMI and eating behaviour but with ambiguous conclusions among relevant studies [47, 48]. A correlation with anorexia nervosa was also observed [49]. It has also been reported that *Methanobrevibacter* and *Christensenella* tend to be highly heritable microbes, exhibiting increased correlation with host genetics [50]. Although phylogenetically distant, they often co-occur in a microbial cooperative (as calculated by co-abundance analysis), as was recently reported for the Russian urban population [51]. Our observations in Yakuts provide additional evidence of potential symbiosis between these two minor but interesting gut taxa. Indeed, a recent co-cultivation study showed that *Christensenella* spp. efficiently support the metabolism of *M. smithii* via H_2_ production [52]*.*

As shown experimentally, *Christensenella* can promote metabolic improvements and is linked to lean body mass [50]. Due to such evidence and several associative studies, this microbe was considered to have high probiotic potential. However, some results show that there is a link between *Christensenella* and CNS disorders, though the link lacks extensive experimental validation. Increased gut levels of *Christensenella* were reported for patients with Parkinson’s disease as well as for multiple sclerosis [8, 13, 53, 54]. In the latter, the proportion of the genus was correlated with the disease severity. *Christensenella* were also observed in a lineage of mice prone to the development of dementia, while the taxon was not detected in the control group [55]. The high heritability of *Christensenellaceae* [56] is particularly interesting in the case of VE, since the risk of VE was shown to be higher for members of a patient’s family.

The trends we observed in microbiome alterations that are linked to VE should be verified in future studies on larger cohorts. Future studies could assess the microbiome’s prognostic potential for VE by considering subjects from the VE risk group. In this pilot study, only one such potential participant was identified and was ultimately excluded due to the intake of oral contraceptives. Intriguingly, when her microbiome composition was analysed with the same protocol used for the other subjects, a high level of *Christensenella* (8.2%) was detected. This result further emphasises the potential interest of this noteworthy gut microbe as a future research topic in the context of the onset and development of VE and other neurodegenerative diseases. In further studies, associations of VE to *Christensenella* and other taxa could be measured on a wider cohort using more cost-efficient targeted tests like taxon-specific gut microbiome qPCR platforms [57].

## Conclusions

This pilot survey of the general Yakut population microbiome shows that its composition combines features that are associated with health and possibly disease risks. The identification of significant shifts in microbiome composition linked to Viliuisk encephalomyelitis even in a small cohort suggests that the disease is likely to involve the microbiome. The possible association of the disease with *Christensenella*—previously linked to potential health benefits—emphasises the complexity of host–microbe interactions and cautions against the introduction of the species as a live biotherapeutic. Surveying vulnerable human populations is a promising approach for mining novel probiotics, preserving indigenous microbiome diversity, and investigating rare diseases and their microbiome-mediated mechanisms and correlates.

## Supplementary information

**Additional file 1**. Clinical data of the participants.

**Additional file 2**. Microbial taxa differentially abundant between healthy Yakuts and residents of Moscow..

## Data Availability

The reads were deposited to the Sequence Read Archive (project ID: PRJNA577062). Basic report for Yakuts, factor report for Yakuts (subjects with and without VE), comparison of VE-free Yakuts with Russian metropolitan population, comparison of VE-free Yakuts with Inuits are available online at: https://biota.knomics.ru/yakuts-ve.

## Abbreviations

BMI: Body mass index
CNS: Central neural system
FMT: Faecal mass transplantation
VE: Viliuisk encephalomyelitis
MRI: Magnetic Resonance Imaging
