# Supplementary material for: Gut microbiome signature of Viliuisk encephalomyelitis in Yakuts includes an increase in microbes linked to lean body mass and eating behaviour
Source: Orphanet J Rare Dis. 2020 Nov 20;15:327. doi: 10.1186/s13023-020-01612-4 (PMC7678198; doi:10.1186/s13023-020-01612-4)
Supplement: Supplementary file 1 — Additional file 1. Clinical data of the participants. [file 13023_2020_1612_MOESM1_ESM.docx]

Supplementary Table 1. Clinical data of participants with definite or suspected chronic VE diagnosis

| **Medical history, symptoms, tests** | **Participant ID** | | | | | | |
| --- | --- | --- | --- | --- | --- | --- | --- |
|  | **V.2** | **V.3** | **V.7** | **V.8** | **V.38** | **V.43** | **V.1^a^** |
| VE diagnosis | Definite | | | | | | Suspected |
| Sex | M | F | M | F | M | F | F |
| Age of onset of VE | 31 | 31 | 24 | 28 | 32 | 48 | 19 |
| Age at the examination | 69 | 48 | 53 | 80 | 59 | 57 | 22 |
| Disease duration (years) | 38 | 17 | 29 | 53 | 27 | 10 | 3 |
| Meningoencephalitic phase | 2 w | 3 w | 2 mo | 1 w | ND | ONMS | ONMS |
| Cranial nerve dysfunction | + | + | + | ++ | + | + | - |
| Bulbar symptoms | - | - | - | + | - | - | - |
| Dementia | - | - | +++ | +++ | ++ | - | - |
| MDRS | 129 | 134 | 116 | 105 | 109 | 128 | ND |
| Dysarthria | ++ | ± | +++ | +++ | ++ | - | - |
| Dysphagia | - | - | + | + | + | - | - |
| Pyramidal signs | +++ | +++ | ++ | +++ | ++ | + | + |
| Babinski sign | +++ | + | +++ | +++ | +++ | ± | **+** |
| Bradykinesia | + | + | ++ | ++ | ++ | - | - |
| Muscle rigidity | **+** | + | ++ | +++ | ++ | - | - |
| Muscle atrophy | + | + | + | ++ | + | - | - |
| Coordination disturbances | ± | - | ± | + | ± | - | ± |
| Sensory disturbances | **-** | - | - | + | - | - | + |
| LMAS | 357 | 378 | 287 | 180 | 298 | 422 | ND |
| MRI: cortical atrophy | **+++** | ND | +++ | +++ | +++ | ND | - |
| Oligoclonal bands in CSF | + | + | + | ND | + | ND | - |

- lack of signs or symptoms;

± mild disturbances;

+ moderate disturbances;

++ pronounced disturbances;

+++ severe disturbances;

ND no data;

w – weeks; mo – months;

MDRS – Mattis Dementia Rating Scale;

LMAS – Lindmark Motor Assessment Scale;

ONMS – organic neurological microsymptoms are considered the onset of the disease.

^a^ Patient V.1 supposedly had acute VE in 2009, history of VE in family, and moderate neurologic disturbances, not sufficient for the definitive diagnosis.
